# The Heart Trumps the Head: Desirability Bias in Political Belief Revision

**DOI:** 10.1037/xge0000298

**Published:** 2017-05-29

**Authors:** Ben M. Tappin, Leslie van der Leer, Ryan T. McKay

**Affiliations:** 1 ARC Centre of Excellence in Cognition and Its Disorders, Department of Psychology, Royal Holloway, University of London; 2 Regent’s School of Psychotherapy and Psychology, Regent’s University London; 3 ARC Centre of Excellence in Cognition and Its Disorders, Department of Psychology, Royal Holloway, University of London

**Keywords:** confirmation bias, desirability bias, motivated cognition, belief updating, politics

## Abstract

Understanding how individuals revise their political beliefs has important implications for society. In a preregistered study (*N* = 900), we experimentally separated the predictions of 2 leading theories of human belief revision—desirability bias and confirmation bias—in the context of the 2016 U.S. presidential election. Participants indicated who they desired to win, and who they believed would win, the election. Following confrontation with evidence that was either consistent or inconsistent with their desires or beliefs, they again indicated who they believed would win. We observed a robust desirability bias—individuals updated their beliefs more if the evidence was consistent (vs. inconsistent) with their desired outcome. This bias was independent of whether the evidence was consistent or inconsistent with their prior beliefs. In contrast, we found limited evidence of an independent confirmation bias in belief updating. These results have implications for the relevant psychological theories and for political belief revision in practice.

People are routinely exposed to a bewildering array of information relevant to their political beliefs. Whether and how they incorporate this information has profound consequences for society. The belief that vaccines have harmful side effects (Moritz, 2011) or that climate change is a hoax (Lewandowsky, Oberauer, & Gignac, 2013) can reduce people’s intentions to vaccinate (Gangarosa et al., 1998; Horne, Powell, Hummel, & Holyoak, 2015; Jolley & Douglas, 2014a) or to minimize their carbon footprint (Douglas & Sutton, 2015; Jolley & Douglas, 2014b). Even simple infographics displayed during live televised election debates can meaningfully shape beliefs about debate outcome, potentially influencing the voting intentions of millions of viewers (Davis, Bowers, & Memon, 2011). A clear understanding of how people incorporate information into their political beliefs is thus of considerable practical importance.

Two prominent theories offer similar yet distinct predictions regarding when and how people incorporate new information into their beliefs. One theory contends that individuals assign greater weight to information that is desirable versus undesirable—that is, a *desirability bias*. This bias is reported to underlie an asymmetry whereby people update their prior beliefs to incorporate new and desirable information more than new but undesirable information (Sharot & Garrett, 2016). The other theory, *confirmation bias,* contends that people preferentially search for, evaluate, and incorporate new information that confirms their prior beliefs (Nickerson, 1998). This bias is reported to underlie an asymmetry whereby people update their prior beliefs to incorporate new and confirming information more than new but disconfirming information—even if they receive a balanced set of both types of information (Lord, Ross, & Lepper, 1979; Taber, Cann, & Kucsova, 2009; Taber & Lodge, 2006).

Unfortunately, the predictions of desirability bias and confirmation bias are often conflated. In the domain of self-belief, the tendency for people to believe desirable things about themselves and their futures (Sedikides & Strube, 1997; Weinstein, 1980) means that new information is typically either confirming and desirable or disconfirming and undesirable (Eil & Rao, 2011). In the domain of political belief, rigorous separation of desirable and confirming information is similarly difficult. Of the few experiments that are appropriately designed to disentangle them, group identity is taken as a proxy for the desirability of information—that is, whether the information is consistent (i.e., desirable) or inconsistent (undesirable) with the position of an individual’s cultural group—and belief updating is not the target outcome measure (e.g., see Kahan, 2016a, 2016b).

Here we experimentally separated desirability bias and confirmation bias in political belief updating. To do so, we capitalized on the political context prior to the 2016 U.S. presidential election. To illustrate the advantage of this context, consider that many supporters of candidate Donald Trump may have believed Hillary Clinton would win the election—owing to her establishment support (Green & Kapur, 2016) or, more conspiratorially, a rigged ballot (Graham, 2016). In such circumstances, new information may have been simultaneously confirming but undesirable (for instance, polls indicating a Clinton win) or disconfirming but desirable (polls indicating a Trump win)—causing desirability bias and confirmation bias to yield divergent predictions for belief updating.

We exploited the profusion of close polling results^1^ to credibly suggest to individuals that either Donald Trump or Hillary Clinton would become the next president and measured how individuals with congruent (i.e., same candidate) desire-belief profiles, and incongruent (different candidate) desire-belief profiles updated their beliefs following receipt of this information. We thus independently manipulated whether information was consistent or inconsistent with (a) who individuals *desired* to win the election or (b) who they *believed* would win the election.

## Method

### Participants

We collected data from 900 participants online via Amazon’s Mechanical Turk (59% female; *M*_age_ = 37.89, *SD* = 12.91). Participants were U.S. residents as determined by IP address (IP addresses located outside the United States were blocked prior to the start of the experiment). We required 779 participants to attain greater than 80% power (α = .05) to detect a small effect of η_p_^2^ = .01 in our primary analyses of covariance. We added approximately 15% to this number to guard against power loss due to planned data exclusions. Following these data exclusions, we retained 811 participants for analyses. The study hypotheses, design, data collection, and analysis plan were preregistered (see https://aspredicted.org/idxgj.pdf).

### Procedure and Design

At the beginning of the survey, participants completed a brief screening questionnaire designed to determine who they (a) *desired* to win and (b) *believed* would win the 2016 U.S. presidential election. Responses to “a” were provided in a nominal choice format: Participants selected *Donald Trump, Hillary Clinton,* or *neither.* Responses to “b” were provided on a bipolar sliding scale from 0 to 100, with *Hillary Clinton* (0) at one end and *Donald Trump* (100) at the other (the numerical values were hidden from participants). Participants were instructed that the more confident they were that a candidate would win, the closer they should slide the pointer to that candidate’s name. Those who responded with scores greater than 50 were categorized as *believing* Trump would win and scores less than 50 as *believing* Clinton would win. Participants selecting *neither* for “a” or exactly 50 for “b” were directed to an end-of-survey message and were unable to continue. This yielded two quasi-experimental groups: those whose *desire-believe* candidates were congruent and those whose *desire-believe* candidates were incongruent. We balanced these condition assignments to obtain approximately 450 in each quasi-experimental condition, with final condition samples after data exclusions as follows: congruent desire–belief: *n* = 406 (desire_Trump_ and believe_Trump_: *n* = 127, desire_Clinton_ and believe_Clinton_: *n* = 279); incongruent desire–belief: *n* = 405 (desire_Clinton_ and believe_Trump_: *n* = 91, desire_Trump_ and believe_Clinton_: *n* = 314).^2^

Participants in both conditions then completed a filler task (the 16-item Balanced Inventory of Desirable Responding; Hart, Ritchie, Hepper, & Gebauer, 2015) before being randomly presented with evidence either consistent or inconsistent with who they believed would win the election. Specifically, participants read a short passage about nationwide polling results, which emphasized either that Hillary Clinton was likely to win the upcoming election or that Donald Trump was likely to win. Participants were also presented with a bar graph figure illustrating such an outcome (study materials are available in the online supplemental materials). Evidence presentation was balanced within each specific candidate that participants initially believed would win the election. For example, of those participants who initially believed Trump would win, half received the polling manipulation suggesting Clinton would win and half received the polling manipulation suggesting Trump would win (likewise for those who initially believed Clinton would win). Thus, collapsing over specific candidates, this yielded four between-subjects conditions in a 2 × 2 design: evidence consistent or inconsistent with who the participant initially believed would win (confirmation: confirmatory or disconfirmatory) and consistent or inconsistent with who they desired to win (desirability: desirable or undesirable). Following the evidence presentation, participants responded to several filler questions about polling data (e.g., “To what extent have you been following the polling data for the upcoming U.S. presidential election?”) before again indicating who they believed would win the election, on the same bipolar scale used initially.

### Belief Updating

We calculated how much participants updated their confidence in who they believed would win the election in the following steps. First, we converted both the participants’ initial confidence (at Time 1 [T1]) and their subsequent confidence (at Time 2 [T2]) into a comparable scale indicating the absolute confidence they had in the candidate they initially believed was most likely to win. Thus, for those who initially believed Trump would win we subtracted 50 from T1 and T2 scores, whereas for those who initially believed Clinton would win we subtracted T1 and T2 scores from 50. Next, we computed the absolute difference between these newly converted T1 and T2 scores for each participant. Finally, we multiplied this difference by either 1 (if the participant updated toward the presented evidence) or −1 (if the participant updated away from the presented evidence), meaning that higher numbers represented greater belief updating toward the presented evidence.

## Results

### Data Exclusions

Participants were excluded from all analyses for fulfilling one or more of these preregistered criteria: failing an attention check embedded in the filler task (*n* = 22; 2.44% of sample), answering “yes” to a question asking them whether they responded dishonestly or mistakenly during the survey (*n* = 48; 5.33%), or recording a belief update score of greater than the mean ± 3 *SD*s in their respective condition (*n* = 26; 2.89%). We excluded one (.11%) further participant for taking the survey more than once (identified via the person’s unique Amazon Mechanical Turk ID). Following these exclusions, 811 participants were retained for analyses.

### Descriptives

**Figure 1 fig1:**
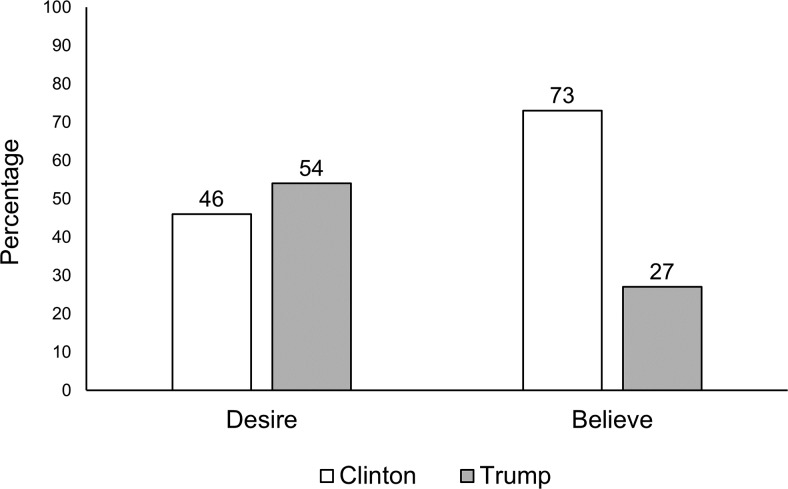
Percentage of participants reporting which candidate they (a) desired to win and (b) initially believed would win the 2016 U.S. presidential election. *N* = 811.

Figure 1 displays the proportion of participants reporting who they (a) desired to win and (b) initially believed would win the election (for these results split by gender, age group, and ethnicity, see Figures S1–S3 in the online supplemental materials).[Fig-anchor fig1]

### Preregistered Analyses

**Figure 2 fig2:**
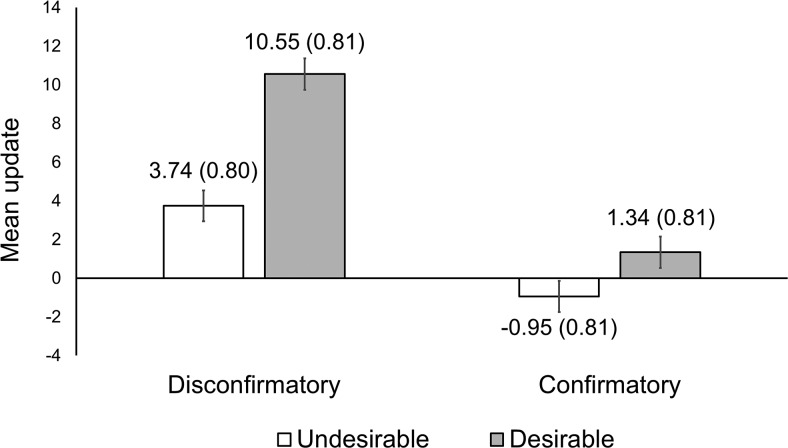
Mean belief update in each between-subjects condition. Error bars and parentheses denote standard error of the mean. Means are adjusted for absolute Time 1 confidence and are based on the 2 × 2 analysis of covariance model. One unit of update corresponds to a 1% adjustment on the bipolar scale used to measure belief. *N* = 811.

We conducted an analysis of covariance (ANCOVA) to investigate the effect of desirability and confirmation on belief updating, adjusting for absolute T1 confidence scores^3^ (Figure 2 displays the adjusted mean update in each condition^4^). There was a main effect of desirability, *F*(1, 806) = 32.81, *p* < .001, η_p_^2^ = .04, 90% confidence interval [CI: .02, .06], such that participants updated more toward the evidence when it was consistent (vs. inconsistent) with the candidate they desired to win. There was also a main effect of confirmation, *F*(1, 806) = 76.63, *p* < .001, η_p_^2^ = .09, 90% CI [.06, .12], but in this case participants updated more toward the evidence when it was inconsistent (vs. consistent) with the candidate they initially believed would win. In other words, we observed a disconfirmation bias. Finally, we observed a small interaction between desirability and confirmation, *F*(1, 806) = 7.15, *p* = .008, η_p_^2^ = .01, 90% CI [.00, .02]. To decompose this interaction, we conducted planned ANCOVAs comparing updating in each condition—while adjusting for absolute T1 confidence scores.[Fig-anchor fig2]

For those participants receiving disconfirming information, updating was greater if that information was desirable (vs. undesirable), *F*(1, 407) = 36.58, *p* < .001, η_p_^2^ = .08, 90% CI [.04, .13]. This pattern was the same for those receiving confirming information, albeit less pronounced, *F*(1, 398) = 20.62, *p* < .001, η_p_^2^ = .05, 90% CI [.02, .09]. Next we examined those participants who received undesirable information; we found that updating was greater for disconfirming (vs. confirming) information, *F*(1, 406) = 23.76, *p* < .001, η_p_^2^ = .06, 90% CI [.02, .09]. This disconfirmation pattern was the same, yet more pronounced, for those receiving desirable information, *F*(1, 399) = 47.72, *p* < .001, η_p_^2^ = .11, 90% CI [.06, .16]. Finally, directly comparing the unique effect of desirable information (disconfirming-desirable condition) against the unique effect of confirming information (confirming-undesirable condition) revealed that updating was greater for the former, *F*(1, 402) = 75.26, *p* < .001, η_p_^2^ = .16, 90% CI [.11, .21].

In the following sections, we report a series of exploratory analyses to examine (a) the robustness of our results and (b) extant debates in the field of politically motivated cognition.

### Robustness

#### Prior exposure

It is likely that participants had different amounts of prior exposure to the election polls. Examination of the distribution of one of our filler questions— “To what extent have you been following the polling data for the upcoming U.S. presidential election?”—suggested this was the case (see Figure S4 in the online supplemental materials). It is possible this affected our manipulation and subsequent results. We thus repeated our preregistered ANCOVA with the addition of this variable as a covariate. However, the pattern of results remained the same.

#### Initial confidence

**Figure 3:**
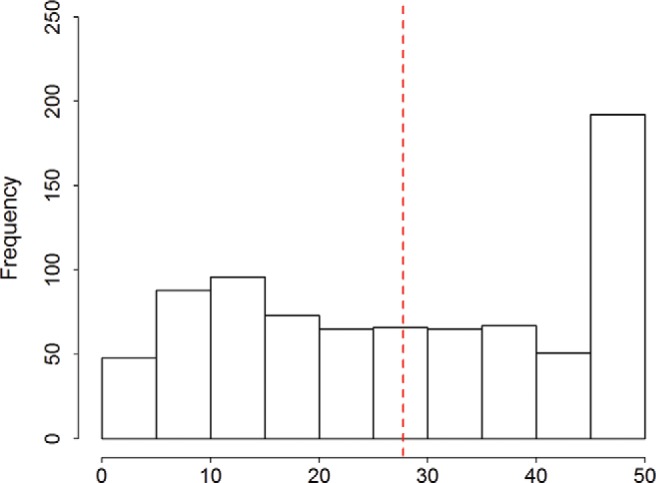
Distribution of absolute Time 1 confidence in belief about which candidate would win the election. The dashed line denotes the median. *N* = 811. See the online article for the color version of this figure.

Participants’ initial (T1) confidence scores were negatively skewed—in particular, a substantial number reported complete (or strong) confidence in their initial belief regarding which candidate would win (see Figure 3). This constrains belief updating for those receiving confirming information because they are unable to update toward the new information (i.e., increase their confidence). In contrast, those receiving disconfirming information can update toward the new information (i.e., decrease their confidence). This may account for the disconfirmation bias we observed.^5^

**Figure 4 fig4:**
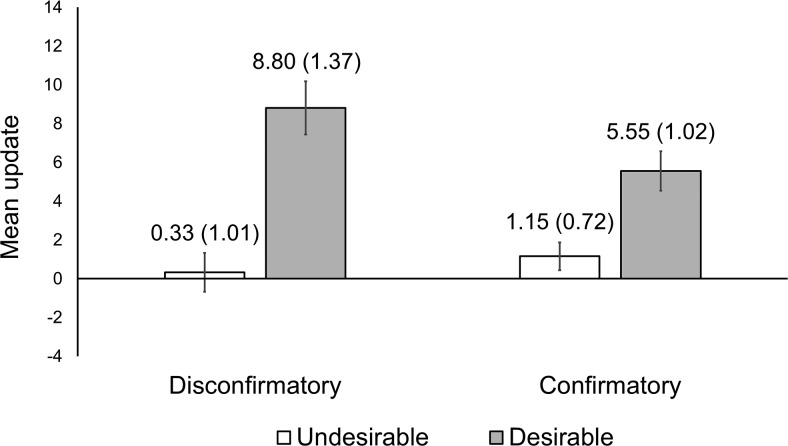
Mean belief update in each between-subjects condition following sample truncation. Error bars and parentheses denote standard error of the mean. Means are unadjusted. One unit of update corresponds to a 1% adjustment on the bipolar scale used to measure belief. *N* = 370.

To explore this possibility, we selected a subset of participants (*n* = 370)—excluding those with high initial confidence (absolute T1 confidence scores >25, *n*_excluded_ = 441)—and recomputed the mean update in each condition (Figure 4 displays the results). The pattern of means in this truncated sample indicated a diminished disconfirmation bias but an enduring desirability bias. To confirm this statistically, we conducted separate Kruskal-Wallis tests on the distribution of belief updating in the confirmation and desirability conditions, respectively.^6^ As suspected, there was now only a trivial difference in updating for participants who received disconfirmatory (*Mdn* = 2.01, interquartile range [IQR] = 11.69) versus confirmatory (*Mdn* = 1.78, IQR = 6.58) information, χ^2^(1, *N* = 370) = 2.01, *p* = .156. In contrast, participants receiving desirable information updated more (*Mdn* = 3.08, IQR = 11.61) than did those receiving undesirable information (*Mdn* = .71, IQR = 6.16), χ^2^(1, *N* = 370) = 25.84, *p* < .001.[Fig-anchor fig4]

To supplement this analysis, we also specifically examined updating among those with weak confidence in their initial belief. This is worthwhile because participants with particularly low confidence may have been (a) less constrained by the upper limit of the confidence scale or (b) simply more receptive to confirming information, compared to their higher confidence counterparts. Thus, we selected those participants with low confidence (absolute T1 confidence scores ≤12.5; *n*_excluded_ = 622) and again recomputed the mean update in each condition. Because the resultant *n* was small (*n*_low confidence_ = 189) and unevenly distributed across conditions, we simulated belief updating scores using the parameters from the low confidence sample. Specifically, for each of the four conditions, we drew 500 scores from a random normal distribution centered on the respective condition mean, as well as the pooled *SD* (i.e., computed across the four conditions; the simulation script and simulated data are available on the Open Science Framework: osf.io/8k92w).

This simulated sample conferred greater than 99% power to detect small effects (η_p_^2^ = .01, α = .05). Conducting an analysis of variance on this data revealed a main effect of desirability, *F*(1, 1996) = 53.73, *p* < .001, η_p_^2^ = .03, 90% CI [.02, .04], similar in size and equivalent in direction to that observed in the preceding empirical analyses. The main effect of confirmation was trivial in size, *F*(1, 1996) = 2.06, *p* = .151, η_p_^2^ = .001, 90% CI [.000, .005], as was the interaction between the two factors, *F*(1, 1996) = 1.54, *p* = .215, η_p_^2^ = .001, 90% CI [.000, .004].

### Ideological Asymmetry Hypothesis

There is ongoing debate over whether motivated cognition is more pronounced among individuals on the political right than the political left (Jost, Glaser, Kruglanski, & Sulloway, 2003; Kahan, 2016b). We thus explored whether supporters of Donald Trump demonstrated greater desirability bias than did supporters of Hillary Clinton. We conducted an ANCOVA (adjusting for absolute T1 confidence as before) with two factors: desirability and a dummy-coded variable denoting which candidate the participant desired to win (“supporter”). There was a small Desirability × Supporter interaction, *F*(1, 806) = 8.58, *p* = .004, η_p_^2^ = .01, 90% CI [.00, .03]. Separate ANCOVA models revealed a stronger desirability bias among supporters of Donald Trump, *F*(1, 438) = 34.07, *p* < .001, η_p_^2^ = .07, 90% CI [.04, .11], than among supporters of Hillary Clinton, *F*(1, 367) = 2.54, *p* = .112, η_p_^2^ = .01, 90% CI [.00, .03].

Further exploration, however, revealed this asymmetry was due to the previously identified ceiling effect in initial (T1) confidence. First, a large number of participants supported Clinton and also believed she would win (*n* = 279), whereas fewer than half this number supported Trump while also believing he would win (*n* = 127). Second, these participants (i.e., those with congruent desire and prior belief) had strong negative skew in their initial confidence, with many believing that their desired candidate was certain to win (see Figure S5 in the online supplemental materials). Taking these facts together implies that supporters of Clinton were more numerous among those who received desirable information but were constrained (by virtue of their extreme initial confidence) in updating their belief toward this information.

This was confirmed by examining participants who (a) had a congruent desire–belief profile, (b) received desirable information, and (c) reported extreme initial confidence (absolute T1 confidence >45). Of these participants (*n* = 69), 67% supported Clinton (*n* = 46) and 33% supported Trump (*n* = 23). This discrepancy may have disproportionately suppressed desirability bias among Clinton supporters. Indeed, truncating the sample to exclude those with extreme initial confidence (absolute T1 confidence >45; *n*_excluded_ = 192, *n*_included_ = 619) and repeating the ANCOVA analysis diminished the size of the Desirability × Supporter interaction, *F*(1, 614) = 1.08, *p* = .298, η_p_^2^ = .002, 90% CI [.000, .012]. Supporters of Donald Trump and supporters of Hillary Clinton demonstrated similar desirability bias in this sample, *F*(1, 348) = 27.19, *p* < .001, η_p_^2^ = .07, 90% CI [.03, .12], and *F*(1, 265) = 10.55, *p* = .001, η_p_^2^ = .04, 90% CI [.01, .08], respectively.

## Discussion

Understanding how people revise their political beliefs has important implications for society. In the context of the 2016 U.S. presidential election, we observed a robust desirability bias: Individuals incorporated information more if it was consistent (vs. inconsistent) with their desired outcome. This bias was independent of whether the information was consistent or inconsistent with individuals’ prior beliefs. In contrast, we found limited evidence of an independent confirmation bias in belief updating. These results have implications for the underlying psychological theories and for political belief revision in practice.

A substantial body of work spanning neuroscience, economics, and clinical psychology has reported an asymmetry in the updating of self-beliefs whereby desirable information is incorporated more than is undesirable information. This asymmetry has been observed when individuals receive information about their personality traits (Korn, La Rosée, Heekeren, & Roepke, 2016; Korn, Prehn, Park, Walter, & Heekeren, 2012), abilities and attractiveness (Eil & Rao, 2011; Mobius, Niederle, Niehaus, & Rosenblat, 2011), or risk of experiencing future negative life events (Moutsiana et al., 2013; Sharot, Korn, & Dolan, 2011; but see Garret & Sharot, 2017; Shah, Harris, Bird, Catmur, & Hahn, 2016). A similar yet distinct asymmetry has been reported in the updating of political beliefs whereby individuals become more confident in their prior beliefs despite receiving a balanced set of confirming and disconfirming information. When two individuals with conflicting prior beliefs are thus exposed to the same stream of information, polarization of political beliefs is an often observed outcome (e.g., Lord et al., 1979; Taber et al., 2009; Taber & Lodge, 2006).

The present study advances this work twofold. First, we found a robust asymmetry in political belief updating that is consistent with desirability bias, independent of individuals’ prior beliefs. In contrast, we found little independent effect of prior beliefs on belief updating. This suggests that the belief polarization reported in previous studies may be due to individuals’ conflicting desires, not their prior beliefs per se. Second, whereas past investigations of political belief updating have mainly focused on political *attitudes* (e.g., support for or against a policy), here we examined belief updating about political *reality*—specifically, individuals’ belief about which presidential candidate was going to be elected. Though one might expect biased belief updating in the former case—after all, attitudes are guided by preferences and desires—it is somewhat more surprising to find that individuals’ desires biased their belief updating over a question of fact (Kahan, 2016a).

A recent study reported that individuals updated their beliefs about the facts of global warming asymmetrically but that the specific pattern depended upon whether they were weak or strong believers in anthropogenic climate change (Sunstein, Bobadilla-Suarez, Lazzaro, & Sharot, 2016). Particularly, when confronted with new information regarding global temperature increase, strong believers updated their beliefs more upon receipt of ostensibly *undesirable* information (i.e., a faster temperature increase than expected), whereas weak believers updated their beliefs more upon receipt of ostensibly *desirable* information (a slower increase than expected). Though this pattern appears consistent with an independent confirmation bias, such an outcome may emerge when individuals are personally invested in “being right”—indeed, for many climate change activists a belief that the world is warming constitutes a core part of their identity (Stern, Dietz, Abel, Guagnano, & Kalof, 1999). For such people, objectively undesirable (but confirming) information about the rate of global warming may be subjectively *desirable,* vindicating their commitment to combatting climate change (Sunstein et al., 2016) and affirming their cultural group identity (Kahan et al., 2012).

It is unlikely that our design inadvertently conflated confirmation with desirability in this way. Ahead of an election, it is difficult to imagine individuals being personally invested in the belief that their desired candidate would *not* get into office. Indeed, in the domain of self-belief updating, rigorous separation of confirming and desirable information yields identical results to those reported in the present study—namely, a robust desirability bias but limited evidence of confirmation bias (Eil & Rao, 2011). We note the important distinction, however, between (a lack of) confirmation bias observed in *belief updating,* as measured here, and confirmation bias, observed in measures of *information search* and *evaluation* (e.g., Ditto & Lopez, 1992). We did not directly examine the latter, which may yet manifest independent of information desirability. Additional exploration of our own data lent support to this distinction (see the Informational Value of Polls section in the online supplemental materials).

Finally, our results offer a mechanistic explanation for why impassioned political disagreements in the United States, such as those over gun control or immigration, appear to be increasingly polarized and intractable (Pew Research Center, 2016). Insofar as individuals have strong preferences concerning these issues (Koleva, Graham, Iyer, Ditto, & Haidt, 2012), our findings suggest they selectively incorporate new evidence into what they believe to be true regarding the relevant facts—provided it is consistent with what they desire to be true. Polarization over factual beliefs is inimical to the effective functioning of a democratic society (Kahan et al., 2012); it is thus a priority to continue exploring which interventions ameliorate the motivated integration of evidence (Lewandowsky & Oberauer, 2016).

## Supplementary Material

10.1037/xge0000298.supp

## Footnotes

1 At the time of study (data collection commenced September 26, 2016; see Real Clear Politics, 2016).

2 The substantial variance in condition sizes per candidate reflects the fact that approximately three quarters of our sample initially believed Clinton would win (see Figure 1 in the Results section)—explaining the smaller number of individuals in the believe_Trump_ condition(s).

3 This prevents regression to the mean spuriously affecting belief updating.

4 The raw means and distributions of update scores are reported in the online supplemental materials.

5 We are grateful to two anonymous reviewers for emphasizing this point.

6 Parametric analyses were inappropriate because the cell *N*s across conditions were unequal following sample truncation.
