# Supplementary material for: The Heart Trumps the Head: Desirability Bias in Political Belief Revision
Source: J Exp Psychol Gen. 2017 May 29;146(8):1143–9. doi: 10.1037/xge0000298 (PMC5536309; doi:10.1037/xge0000298)
Supplement: Supplementary file 1 [file zfr999172920so1.docx]

**Supplemental Materials**

**The Heart Trumps the Head: Desirability Bias in Political Belief Revision**

**by B. Tappin et al., 2017, *Journal of Experimental Psychology: General***

**http://dx.doi.org/10.1037/xge0000298**

***Screening Questionnaire.***

Participants completed the following questions as part of the screening procedure (in a fixed order):

1. *Please enter your age (in years):*
2. *Please select your gender:*
   1. *Female*
   2. *Male*
   3. *Other*
3. *Please select your ethnicity:*
   1. *Asian*
   2. *Black*
   3. *Hispanic*
   4. *White*
   5. *Other (please enter):*
4. *Please indicate your religious affiliation:*
   1. *Agnostic*
   2. *Atheist*
   3. *Christian*
   4. *Jewish*
   5. *Muslim*
   6. *Other (please enter):*
5. *Which political candidate do you* ***want*** *to win the upcoming US presidential election?*
   1. *Donald Trump*
   2. *Hillary Clinton*
   3. *Neither*
6. *Which political candidate do you****think****will win the upcoming US Presidential election?*

*Please provide your response using the sliding scale below (****note:****dragging the slider closer towards the name of the candidate indicates how confident you are).*

Hillary Clinton |------------------------------------**◊**------------------------------------| Donald Trump

***Filler questions.***

After the screening questionnaire, those who were eligible to continue with the survey completed the *16-item balanced inventory of desirable responding* (BIDR, Hart et al., 2015). Completed on a scale from 1 (Strongly disagree) to 7 (Strongly agree). The question order was fixed.

***Polling Manipulation.***

Following the 16-item BIDR, participants (according to condition) read the following passage:

*Over the past several months there have been many polls conducted to try and predict the outcome of the upcoming US Presidential election: specifically, whether it will be Hillary Clinton or Donald Trump who assumes the mantle of commander-in-chief.*

*These polls are conducted at both the local (i.e., state-wide) and nationwide level. As Election Day draws closer, several of the large nationwide polls become increasingly indicative of who will go on to win the election and become the next President of the United States. Examples of such nationwide polls are the USC/Los Angeles Times and NBC/Survey Monkey tracking polls, the Google Consumer Surveys poll, and the Ipsos/Reuters Core Political Data survey.*

*As you may be aware, data from several of these nationwide polls have recently suggested that, on Election Day, Hillary Clinton* [Donald Trump] *is likely to obtain the largest proportion of votes, and thus be elected as President of the United States. The results of nationwide polling data are not definitive, but results this close to Election Day have proven accurate in correctly identifying the election of Presidential candidates in the past.*

Participants in the Clinton-win condition saw the following graphic:


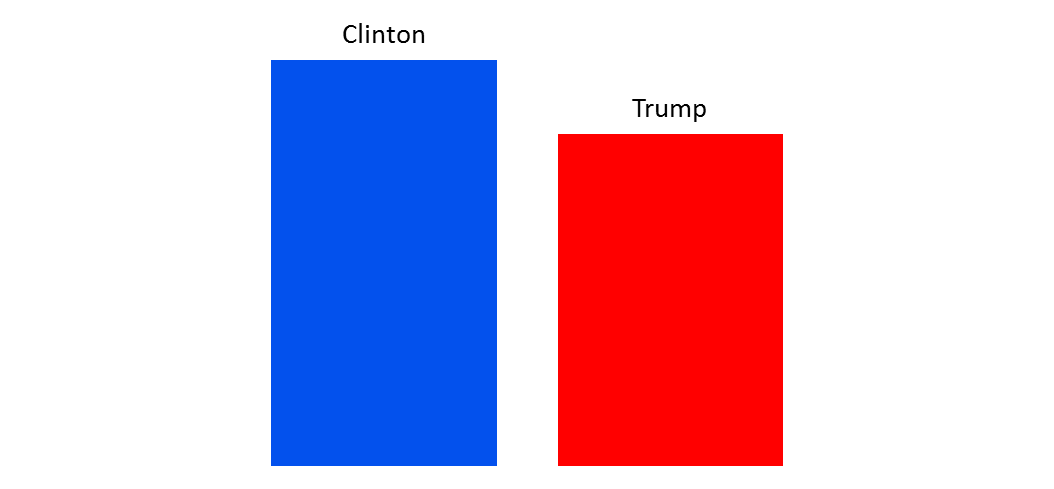


Participants in the Trump-win condition saw the following graphic:


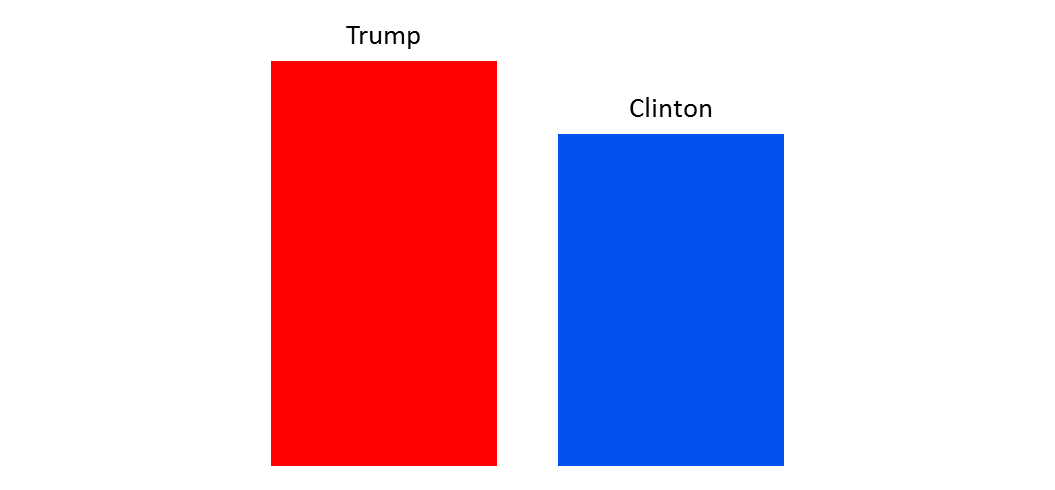


***Polling data (filler) questions*.**

Following the manipulation, participants responded to the following three filler questions (fixed question order):

1. *In general, do you think polling data is informative?* [Scored from 1 (Not at all) to 7 (Very much so)]
2. *Do you think there should be more or less polling data made available to the public prior to US presidential elections?* [Scored from 1 (Definitely less) to 7 (Definitely more)]
3. *To what extent have you been following the polling data for the upcoming US presidential election?* [Scored from 1 (Not at all) to 7 (Very much so)]

***Time 2 belief.***

Participants then once again indicated who they believed would win the election (on the same bipolar scale used previously):

*Finally, given these nationwide polling data and your own opinion, please indicate which political candidate you****think****will win the upcoming US Presidential election.*

*Please provide your response using the sliding scale below (****note:****dragging the slider closer towards the name of the candidate indicates how confident you are).*

Hillary Clinton |------------------------------------**◊**------------------------------------| Donald Trump

***Final questions.***

On the last page of the survey participants were asked:

*Accurate data are very important for our research so please answer the following question honestly.*

*Your answer to this question is completely anonymous, and we can assure you that there will be no negative consequences whatsoever for answering "yes".*

*You will get your HIT code on the next screen regardless of how you respond, and your response will have no influence on what Mechanical Turk HITs you can choose to do in the future.*

*During this survey, did you answer dishonestly or mistakenly at any point?*

1. *Yes 2. No*

*Lastly, we would like to hear any feedback you have about the survey. Please leave any feedback in the box below. If you have no feedback, please enter "none" into the box.*
