# Supplementary material for: The Heart Trumps the Head: Desirability Bias in Political Belief Revision
Source: J Exp Psychol Gen. 2017 May 29;146(8):1143–9. doi: 10.1037/xge0000298 (PMC5536309; doi:10.1037/xge0000298)
Supplement: Supplementary file 2 [file zfr999172920so2.docx]

**Supplemental Materials**

**The Heart Trumps the Head: Desirability Bias in Political Belief Revision**

**by B. Tappin et al., 2017, *Journal of Experimental Psychology: General***

**http://dx.doi.org/10.1037/xge0000298**

**Supplement: Analyses**

**Descriptives**

Figures S1-S3 (below) show whom participants (a) desired to win and (b) initially believed would win the 2016 US presidential election, by gender (Figure S1), age group (Figure S2) and ethnicity (Figure S3).

*Figure S1. Percentage of males and females reporting which candidate they (a) desired to win and (b) initially believed would win the 2016 US presidential election. Please note: N=810. One participant did not identify as male or female; they reported desiring Trump but believing Clinton would win.*

*Figure S2.* *Percentage of participants in each age group (in years) reporting which candidate they (a) desired to win and (b) initially believed would win the 2016 US presidential election. Note: <30 n=265; 30-40 n=250; 40+ n=296. Total N=811.*

*Figure S3.* *Percentage of Non-White and White participants reporting which candidate they (a) desired to win and (b) initially believed would win the 2016 US presidential election. Note: Non-White n=152; White n=659. Due to the low number of Non-White participants we pooled them into a single category to provide an interpretable summary.*

**Robustness**

*Prior exposure.*


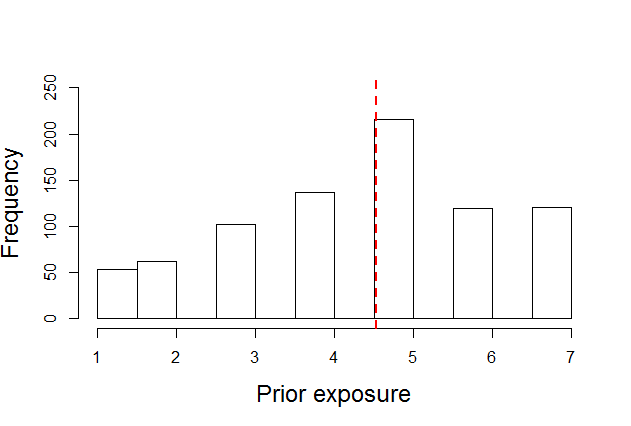
Figure S4 (below) shows the distribution of scores on the filler question “*To what extent have you been following the polling data for the upcoming US presidential election?* [Scored from 1 (Not at all) to 7 (Very much so)].

*Figure S4.* *Distribution of scores indicative of prior exposure to US election polls. Note: Scored from 1 (Not at all) to 7 (Very much so); the dashed line denotes the mean. N=811.*

**Ideological Asymmetry Hypothesis**

Figure S5 (below) shows the distribution of initial (T1) confidence in the candidate participants believed would win the election, crossed with whom they desired to win the election.


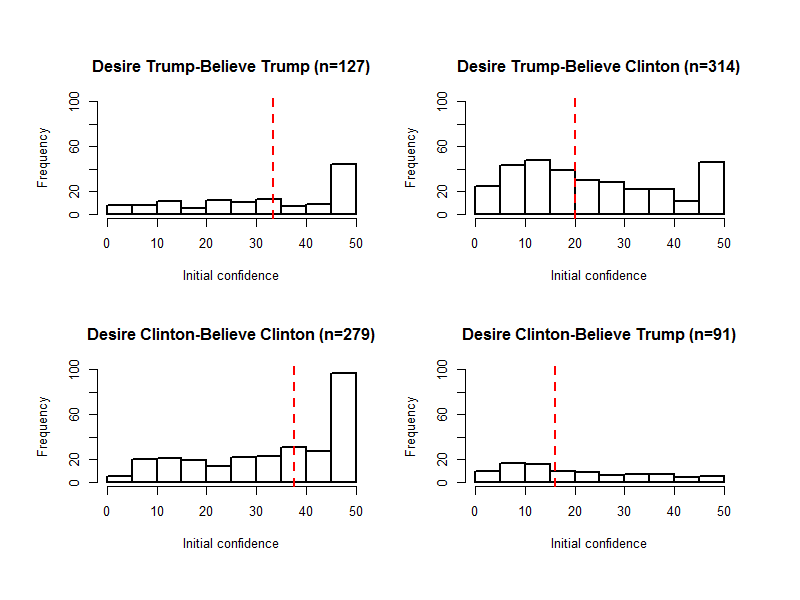


*Figure S5.* *Distribution of absolute T1 confidence according to which candidate participants (a) desired to win, and (b) initially believed would win. Note: The dashed line denotes the median. N=811.*

**Additional exploratory analyses**

***Informational value of polls.***

We examined the effect of Confirmation and Desirability on participant responses to one of our filler questions— “*In general, do you think polling data is informative?*” [Scored from 1 (Not at all) to 7 (Very much so)]. Conducting a two factor ANOVA revealed a main effect of Desirability, F (1, 807) = 28.86, p <.001, η_p_² = .04 90% CI [0.02, 0.06], such that participants thought polling data was more informative when it was consistent (M = 4.95, SD = 1.40) versus inconsistent (M = 4.41, SD = 1.56) with whom they desired to win the election. We also observed a main effect of Confirmation, F (1, 807) = 65.86, p <.001, η_p_² = .08 [0.05, 0.11], such that participants thought polling data was more informative when it was consistent (M = 5.08, SD = 1.42) versus inconsistent (M = 4.27, SD = 1.48) with whom they initially believed would win the election. There was only a trivial interaction between the factors, F (1, 807) = 0.01, p =.933, η_p_² < .001.

We also investigated to what extent supporters of Donald Trump and supporters of Hillary Clinton differed in their judgments of the informational value of polling data overall. An independent samples t-test revealed a trivial difference between supporters of Trump (M = 4.61, SD = 1.58) and supporters of Clinton (M = 4.75, SD = 1.42): t (809) = 1.31, p =.192, d = 0.09 95% CI [-0.05, 0.23].

***Participants who changed their belief.***

Of the full sample (n=811), 62 (7.64%) changed their (qualitative) belief about which candidate was going to win the election. This was determined by examining the sign of absolute T2 confidence: if participants crossed the midpoint of the bipolar scale when giving their belief for a second time (i.e., indicating they now believed that a different candidate was most likely to win the election) the sign would be negative. As to be expected, participants who changed their belief had lower confidence in their initial belief (M_T1 confidence_ = 15.66, SD_T1 confidence_ = 12.42) than those who did not change their belief (M_T1 confidence_ = 29.15, SD_T1 confidence_ = 15.81). Participants who changed their belief also had much larger belief updating scores (M_update_ = 25.10, SD_update_ = 23.02) than those who did not (M_update_ = 1.92, SD_update_ = 8.61).

Of those who changed their mind, half were supporters of Donald Trump (n=32, 7.26% of all Trump supporters), and half were supporters of Hillary Clinton (n=30, 8.11% of all Clinton supporters). Of those who initially believed Clinton was most likely to win (n=593), 32 (5.40%) subsequently believed Trump was most likely to win; whereas, of those who initially believed Trump (n=218), 30 (13.76%) changed their belief to Clinton. Finally, receiving the polling manipulation which suggested that Trump was going to win (n=414) prompted 40 (9.66%) individuals to change their belief about which candidate was most likely to win; whereas only 22 (5.54%) of those who received the Clinton manipulation (n=397) did the same. This was probably a function of the fact that approximately three-quarters of the sample initially believed Clinton was more likely to win.

**Raw means and distributional information**

Table S1 presents the raw means and standard deviations of update scores by experimental condition. The distributions of these scores are displayed in Figure S6.

Table S1. Raw means and standard deviations of update by experimental condition.

| **Condition** | **M** | **SD** |
| --- | --- | --- |
| Disconfirmatory-Undesirable | 3.58 | 9.77 |
| Disconfirmatory-Desirable | 10.71 | 16.39 |
| Confirmatory-Undesirable | -0.80 | 9.28 |
| Confirmatory-Desirable | 1.19 | 7.78 |
| *Note: Total N=811.* | | |


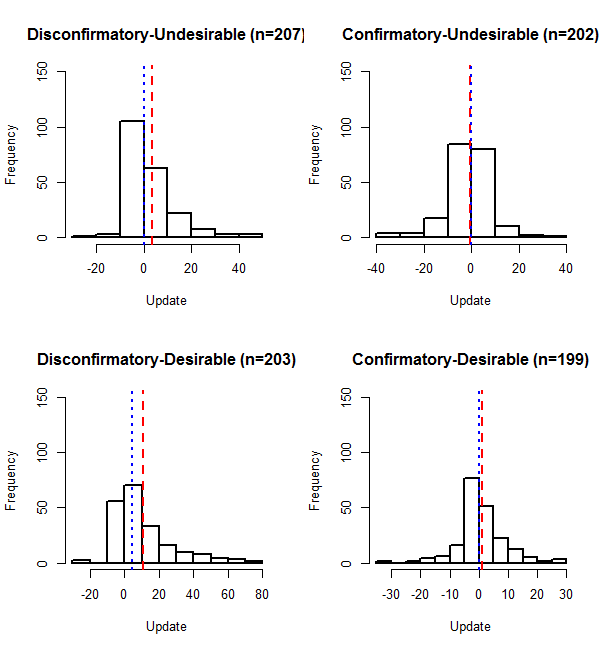


*Figure S6.* *Distribution of update scores according to condition. Note: The larger dashed line denotes the mean and the smaller dashed line denotes the median. Total N=811.*

***Skewed update scores.***

Update scores in the Disconfirmatory-Desirable and Disconfirmatory-Undesirable conditions were positively skewed (see Figure S6), which may have spuriously generated the Desirability and (dis) Confirmation biases observed in the full sample (N=811), respectively. To rule out this possibility, we conducted separate Kruskal-Wallis tests on the distribution of updating in the Desirability and Confirmation conditions in the full sample. These analyses revealed that updating was greater for desirable information (Median = 1.90, IQR = 9.79, n = 402) than undesirable information (Median = 0.00, IQR = 6.61, n = 409), χ² (1, N = 811) = 22.15, p <.001. Similarly, updating was greater for disconfirmatory information (Median = 1.90, IQR = 11.37, n = 410) than confirmatory information (Median = 0.00, IQR = 5.69, n = 401), χ² (1, N = 811) = 42.23, p <.001. The results of these nonparametric analyses thus mirror the results of the pre-registered analyses reported in the main text.
